# Implementing multiple implicit association tests in Qualtrics: A guide and demonstration using balanced identity design theory

**DOI:** 10.3758/s13428-026-03047-4

**Published:** 2026-05-29

**Authors:** Alyssa Ream, Anna Woodcock, Sarah Zlatkovic, Rachelle M. Pedersen, Ashley Bonilla, Hannah Middleton, Paul R. Hernandez, P. Wesley Schultz

**Affiliations:** 1 Claremont Graduate University, 150 E 10th St, Claremont, CA 91711 USA; 2 Texas Tech University, 2500 Broadway, Lubbock, TX 79409 USA; 3 Texas A&M University, 400 Bizzell St, College Station, TX 77843 USA

**Keywords:** IAT, Implicit associations, Implicit identities, Qualtrics, Data collection, Online research

## Abstract

Interest in implicit processes, such as attitudes and identities, has grown in behavioral sciences since Greenwald and colleagues (1998) developed and introduced the Implicit Association Test (IAT). The IAT was developed to measure these processes, which has become more accessible with the popularity of Qualtrics as a data collection tool, especially through methods and code provided by Carpenter et al. (2019). They demonstrated how to construct a single IAT for use on the Qualtrics platform, providing research resources for creating IATs to examine concept-pair associations. However, there is no guidance on constructing multiple IATs on survey software platforms for research that requires two or more IATs in a single study. This paper extends Carpenter et al.’s (2019) work by outlining the process of developing, implementing, and utilizing multiple IATs within a single Qualtrics survey. We provide a tutorial using examples from our research using the Balanced Identity Design (BID) framework, including step-by-step written and visual instructions and templates, instructions for planning and building multiple IATs and dynamically presenting them in a single Qualtrics survey and code for processing IAT data. We also demonstrate the utility of the multiple IAT approach by reporting a short study utilizing BID theory wherein we measure implicit racial/ethnic identity, STEM identity, and race/ethnicity-STEM associations in a single study via three IATs.

## Abstracta

El interés en los procesos implícitos, como las actitudes y las identidades, ha crecido considerablemente en las ciencias del comportamiento desde que Greenwald y sus colegas (1998) introdujeron el Test de Asociación Implícita (IAT). El IAT fue diseñado para medir estos procesos, y su uso se expandió a medida que los investigadores adoptaron Qualtrics como herramienta de recolección de datos, en particular a través de los métodos y el código compartidos por Carpenter et al. (2019). Ese trabajo mostró cómo construir un solo IAT dentro de Qualtrics, dándoles a los investigadores un punto de partida concreto para estudiar asociaciones entre pares de conceptos. Lo que no abordó fue cómo construir y administrar múltiples IATs en un solo estudio dentro de plataformas de encuestas. Este artículo retoma el trabajo de Carpenter et al. (2019). Describimos el proceso de desarrollar, configurar y utilizar múltiples IATs dentro de una sola encuesta en Qualtrics. Usando nuestra propia investigación con el marco de Diseño de Identidad Balanceada (BID) como guía, ofrecemos un tutorial paso a paso con instrucciones escritas, elementos visuales y plantillas. Cubrimos cómo planificar y construir múltiples IATs, cómo presentarlos de forma dinámica dentro de una sola encuesta, y cómo procesar los datos una vez recolectados. También reportamos un estudio breve usando la teoría BID para ilustrar lo que este enfoque hace posible: en un solo estudio, medimos la identidad de género implícita, la identidad en STEM y las asociaciones entre género y STEM mediante tres IATs.

## Introduction

Interest in implicit processes such as attitudes, self-esteem, identities, and cognitive associations between constructs has steadily grown in behavioral sciences research since Greenwald and colleagues (1998) developed the IAT. Developing the Implicit Association Test (IAT) was a pivotal advancement in measuring these implicit processes. The IAT assesses the strength of associations between mental concepts by having individuals classify stimuli as quickly and accurately as possible into different categories. Its effectiveness in measuring association strength depends on the assumption that when two concepts are strongly associated, the sorting task is significantly easier than when they are weakly associated (Greenwald et al., 2002).

The measurement of implicit processes has become increasingly accessible to researchers, aided in part by the growing popularity of Qualtrics (Qualtrics, 2024) as a data collection tool and open access to methods and code like the resources provided by Carpenter et al. (2019), Millisecond.com, and Greenwald’s website. Carpenter and colleagues demonstrated how to construct a single IAT for use on the Qualtrics online survey platform, providing resources for researchers to create their own IAT for examining single concept-pair associations, such as implicit self-esteem (e.g., me = good, me = bad) or identity (e.g., me = math, me = liberal arts), stereotypes, (math = male, nurturing = female), or attitudes and prejudices (e.g., White = good, overweight = bad). However, there are few tutorials in the literature on constructing *multiple* IATs in survey software platforms, such as Qualtrics, for researchers who need to run multiple IATs in a single study.

This paper extends Carpenter et al.’s (2019) work by presenting a method for developing, implementing, and utilizing any number of IATs within a single online Qualtrics survey, adaptable to any multi-IAT design. The provided step-by-step instructions and reusable code not only enable researchers to dynamically present, and randomize any number of IATs, but also ensure data validity through IAT accuracy calculations and recording the order of IAT encounters. To illustrate this method in application, we use the example of Balanced Identity Design (BID) Theory research, which requires multiple participant-tailored IATs to be randomized and dynamically presented within a single study to measure implicit identities and associations (Cvencek et al., 2012; Greenwald et al., 2002; Woodcock et al., 2025), without compromising reliability or validity.

## Section 1: The multiple IAT method

This paper begins by providing an overview and in-depth explanation of the procedures for developing and implementing multiple IATs, extending the work of Carpenter et al. (2019). Afterward, a short empirical study is presented to demonstrate the application of multiple IATs within a single study of racial/ethnic disparities in STEM.

Creating multiple IATs in a single Qualtrics survey or data collection tool requires a working knowledge of R and R Studio (R Core Team, 2023; RStudio Team, 2020), building and deploying surveys in Qualtrics, and collecting and retrieving data from Qualtrics. Some specific Qualtrics skills that should be mastered before attempting to build multiple IATs are:

- Building a basic survey
- Understanding blocks
- Understanding survey flow (e.g., branching) and embedded data
- Using e-mail triggers

Some specific skills that should be mastered in R and R Studio are:

- Basic R coding literacy
- Scripting and customizing R scripts
- Working with R packages
- Troubleshooting code

There are many excellent online resources for learning Qualtrics, including the extensive help section and community (see https://community.qualtrics.com).

**Fig. 1:**
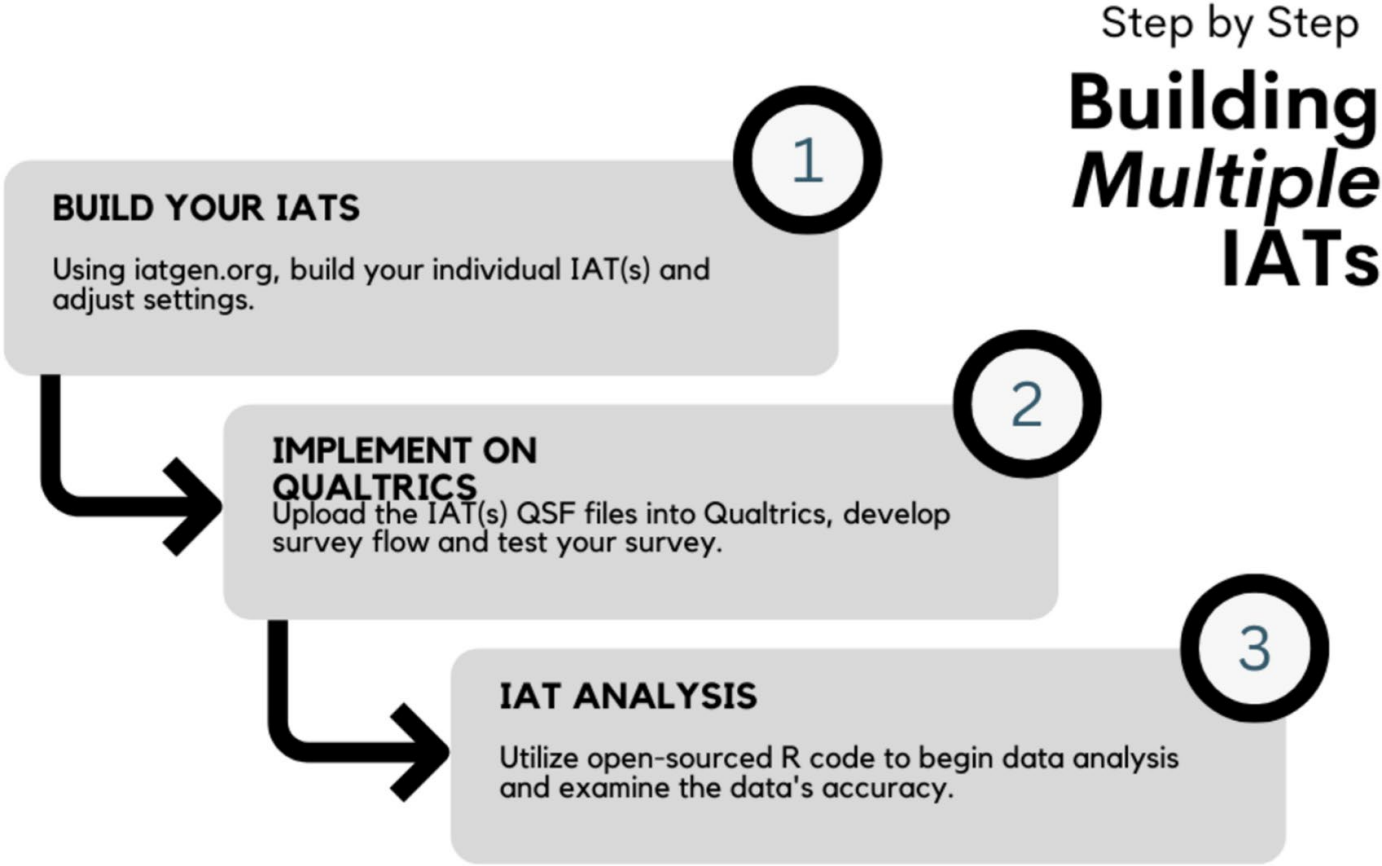
Simplified step-by-step process for building multiple IATs

We present a step-by-step guide for *1)* building a series of multiple IATs, *2)* implementing and situating multiple IATs in Qualtrics, and *3)* calculating the IAT D-scores and analyzing the IAT data. Readers who are familiar with building IATs in *iatgen* or who have existing IATs may begin with Step 2, where the instructions for implementing multiple IATs in Qualtrics begin. Those who are unfamiliar with building and implementing IATs should begin with Step 1, and it is recommended to review the overviews and tutorials outlined by Carpenter et al. (2019). See Fig. 1 for an illustration of the steps.

The IAT method utilizes:

- Iatgen webpage—http://iatgen.org/ that’s within https://iatgen.wordpress.com^1^
- Shiny—a package that makes building interactive web apps straight from R and Python easy: https://shiny.posit.co/ (Chang et al., 2026)
- Qualtrics – https://www.qualtrics.com/ an online platform for building and distributing surveys
- R—an open-source statistical analysis and data visualization programming language
- RStudio—an integrated development environment for R

We will illustrate the process of creating, testing, deploying, and managing data for multiple IATs using a fictitious example from our research program using Balanced Identity Design to address issues of underrepresentation of women in STEM in the United States. The example consists of multiple IATs presented within a Qualtrics survey whereby participants click on the survey link, are presented with an informed consent screen, several survey items, three randomly pre-inserted IATs catered to their specific STEM major, and separated by instruction screens, and then a longer battery of survey items. Please note that the multiple IAT processes described here relate to IATs taken on a laptop or desktop (*not* a tablet or smartphone). We have included supplemental online material that describes the process of setting up Qualtrics to identify and respond to participants who attempt to start the IATs on a tablet or smartphone (see SOM 1).

### Demonstration

Our step-by-step demonstration for building, presenting, and processing data for multiple IATs in a single study will use an example of STEM-gender identity balance in a sample of male and female engineering, science, and computer science majors who were classified by the survey questions “Which gender do you identify with the most strongly?” and “Which is your current college major?” This yielded six classifications of participants:

1. Male—science major
2. Female—science major
3. Male—engineering major
4. Female—engineering major
5. Male—computer science major
6. Female—computer science major^2^

**Fig. 2:**
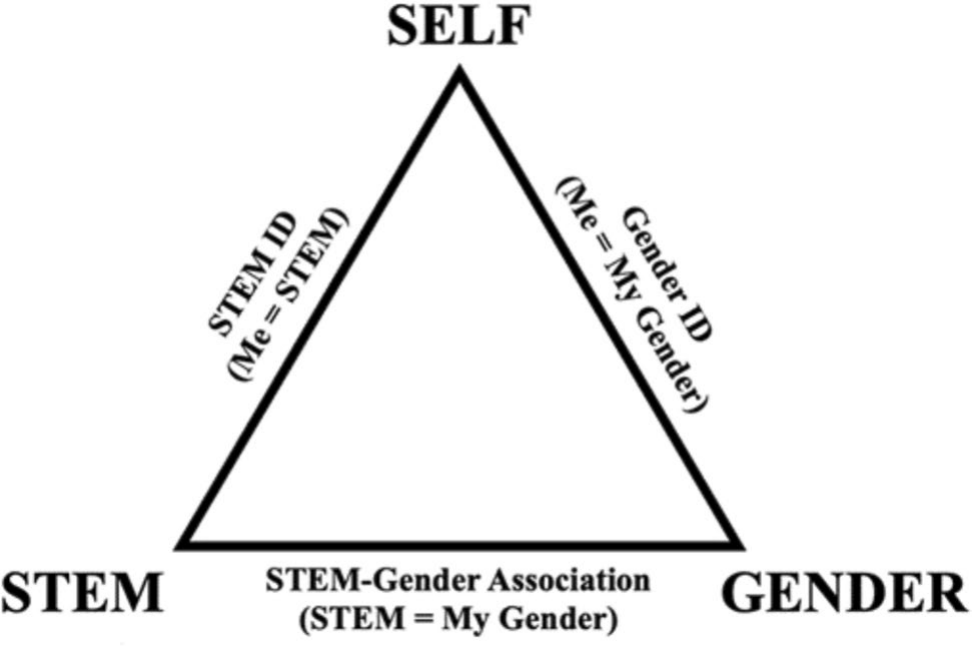
Balanced identity design framework

Balanced Identity Design measures the psychological balance or imbalance between three implicit associations or identities. The current example illustrates how to create and implement multiple IATs to collect data about the identity balance of men and women in STEM majors. In this instance, BID requires collecting IAT data for two identities: STEM domain identity (me = science, me = engineering, or me = computer science) and gender identity (me = male, or me = female). In addition, we collected implicit gender-STEM associations (e.g., male = engineering, female = engineering, etc.). Each participant received a dynamic, personalized combination of three of 11 possible IATs (three STEM identities, two gender identities, and six possible gender-STEM associations). See Fig. 2 for a visual of the identity balance configuration for gender and STEM.

### Step 1: Build individual IATs

The first step is to build the required number of individual IATs on http://iatgen.org/ before building the entire survey in Qualtrics. Using Carpenter and colleagues’ (2019) method, using the http://iatgen.org/ webpage, the researcher can create each IAT – 11 in total in this example. Many customizable parameters must be considered and are explained in the following sections. For this example, 11 IATs must be created to measure gender identity, STEM identity, and gender-STEM associations. Navigate to the http://iatgen.org/ webpage. The researcher will have the option to “Setup IAT,” “Analyze IAT,” or “Cite iatgen.” To build the IATs, navigate to the “Setup IAT” option.

#### IAT name

Prior to setting up the first of the IATs, it is *essential* to identify a unique name for each IAT – in our example:

1. Female Gender Identity IAT (e.g., Me = Female)
2. Male Gender Identity IAT (e.g., Me = Male)
3. Engineering Identity IAT (e.g., Me = Engineering)
4. Science Identity IAT (e.g., Me = Science)
5. Computer Science Identity IAT (e.g., Me = Computer Science)
6. Male-Science Association IAT (e.g., Science = Male)
7. Female-Science Association IAT (e.g., Science = Female)
8. Male-Engineering Association IAT (e.g., Engineering = Male)
9. Female-Engineering Association IAT (e.g., Engineering = Female)
10. Male-Computer Science Association IAT (e.g., Computer Science = Male)
11. Female-Computer Science Association IAT (e.g., Computer Science = Female)^3^

Properly labeling IATs reduces confusion and potential errors when importing them into a survey. The label names will also be used to label embedded data fields and record trial responses in Qualtrics. Once the researcher has given the IAT a name—in this case, we will build a “Female Gender Identity IAT”—the researcher will continue to add the attribute and target stimuli.

#### Attribute, target, stimuli, and type

**Fig. 3:**
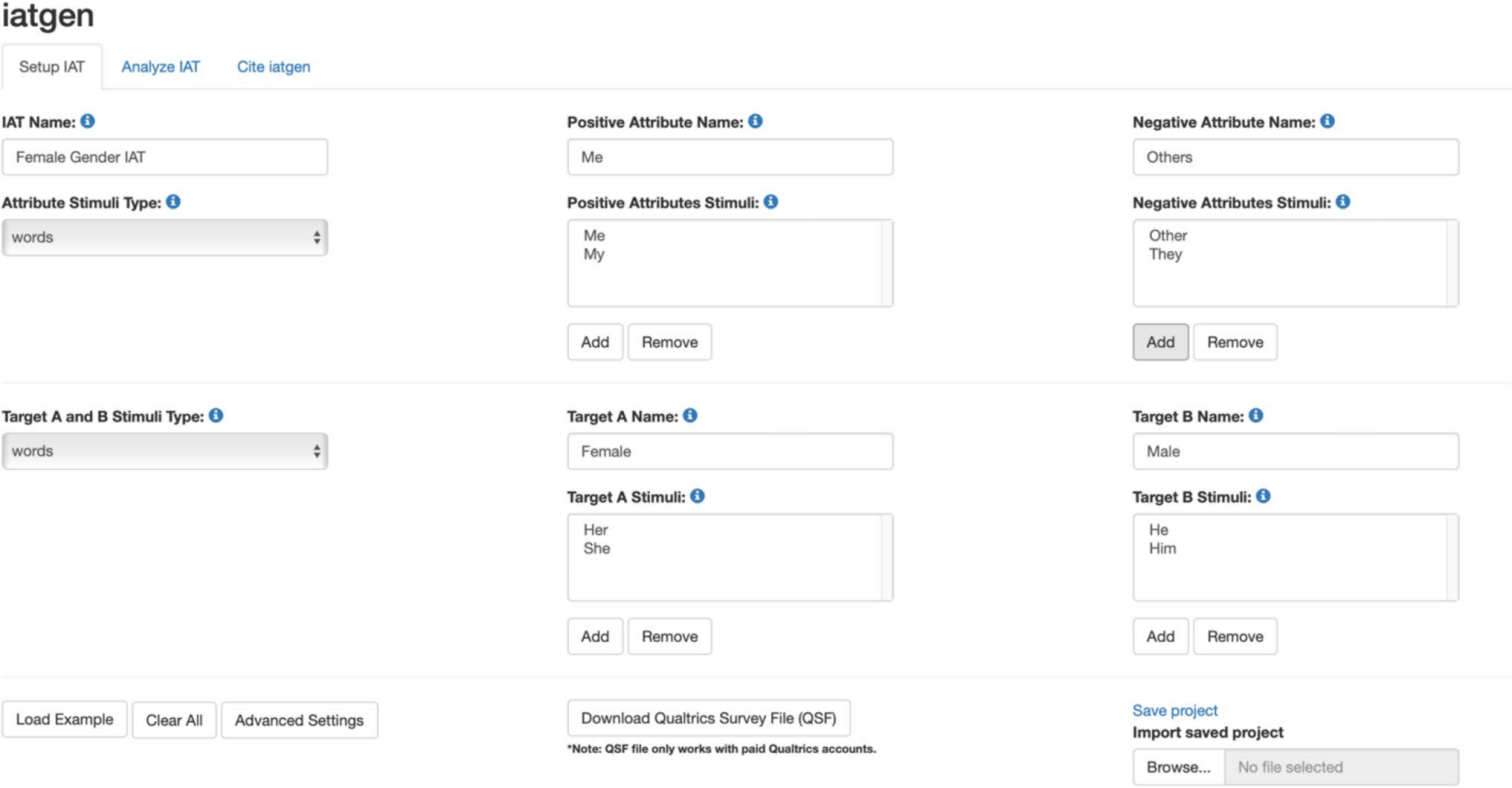
Example attribute and target stimuli set up on http://iatgen.org/. *Note.* Positive Attribute and Target A are stimuli that are *compatible* with each other. On the other hand, Negative Attribute and Target B are stimuli that are *incompatible* with each other. This means that if the researcher wants to measure the IAT relationship, such as Female gender identity, Female and Me will be placed in the Positive Attribute and Target A sections

On the http://iatgen.org/ webpage, there are boxes to input the IAT categories and stimuli. There are four categories of stimuli: two for the targets and two for attributes, which can be text or images. In this example, we utilize word stimuli rather than images.^4^ Input the stimuli into the corresponding boxes. For example, to develop a Female Gender IAT, Target A and B attributes would include “Female” and “Male” with stimuli including items such as the words “her,” “he,” “she,” and “him.” While the positive and negative attribute categories may consist of “Me” and “Others” with stimuli that might include words such as “them,” “me,” and “they.” See Fig. 3.

#### Advanced settings

Once stimuli and categories have been selected, under advanced settings, researchers can customize the presentation of the IAT, including word color, number of trials, pause time, and error pauses. We provide a template in the supplementary materials (See SOM 2). To import the template, go to http://iatgen.org/, then on the bottom right-hand corner, under “Import saved project,” select “Browse,” and choose the JSON template file. Stimuli color and error pauses can be further tailored. However, this template-setting decision is based on Carpenter and colleagues' (2019) settings, as they reported empirical findings on reaction times. Once an IAT is finalized, the investigator can download the IAT as a QSF (Qualtrics survey file) by pressing “Download Qualtrics Survey File (QSF)” on the webpage; this can be easily imported into Qualtrics later. To create multiple IATs, repeat this process for however many IATs are desired.

### Step 2: Implementing multiple IATs in qualtrics

**Fig. 4:**
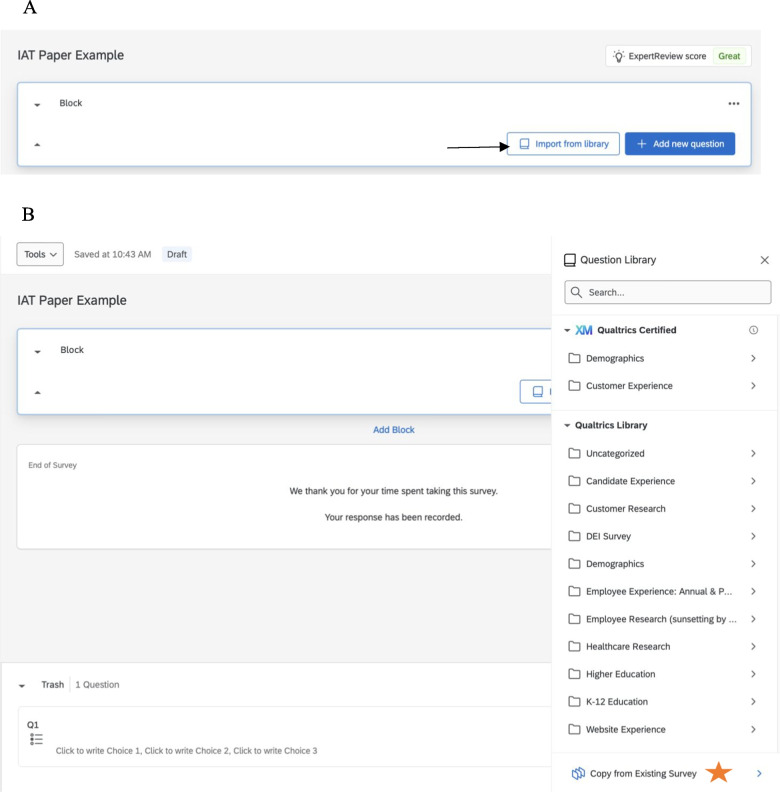

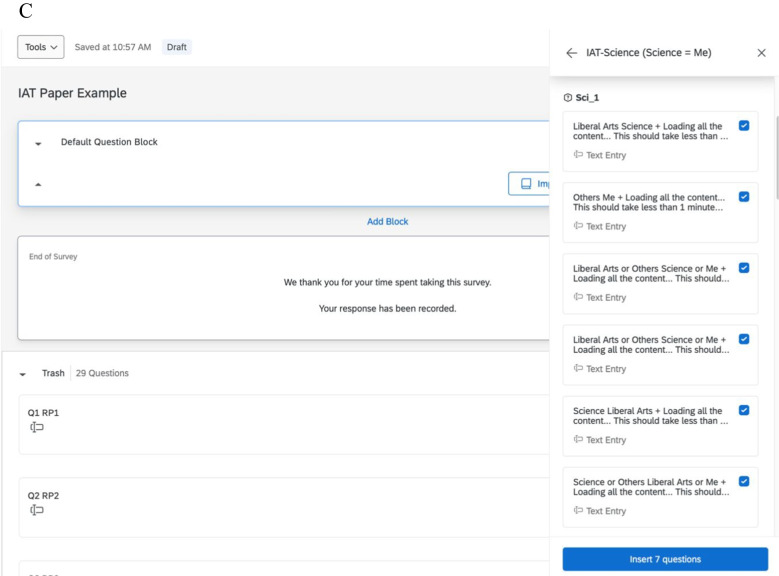
Panel A—*Location of import from library. Note.* Within the Qualtrics survey, create a new block, and select “import from library.” Panel B—*Location of copying from existing survey. Note.* Once “import from library” is selected, select “copy from existing survey.” Then, navigate to one of the created IATs and transfer each of the four blocks of the one IAT into their own blocks on the new survey. Panel C—*Inserting IAT blocks into existing survey. Note.* To import the first block of the IAT, select it (e.g., Sci_1) and then insert seven questions into the block. After that, create a new block and insert the next seven questions (i.e., Sci_2). Repeat this process until all four blocks have been imported (i.e., Sci_1, Sci_2, Sci_3, Sci_4) into their own blocks of the IAT into the new survey. Keep in mind that this process is only for importing one IAT. If there are multiple IATs, this process will need to be repeated for each one

Until now, we have been following the procedures set forth by Carpenter et al. (2019), but this is the point of divergence for investigators needing to present two or more IATs in a single Qualtrics survey. The process we describe assumes the researcher has a survey pre-built in Qualtrics, for which we will add IAT surveys. In our example, we have a survey with a welcome screen (Block 1), an informed consent page (Block 2), and some survey items (Block 3), followed by more survey items (Block 5), and a thank you screen (Block 6). The IATs will be embedded between Blocks 3 and 5. The IATs can functionally appear anywhere in an existing survey that the researcher wishes. We chose to embed our IATs between the first and second set of survey items so *1)* the IATs are presented toward the beginning of the survey, increasing the likelihood of completion, and *2)* the survey items that trigger the IAT selection are presented to participants before they complete their IATs. The process of embedding multiple IATs is as follows:

1. Create a folder in Qualtrics to house the IAT surveys.
2. Create a blank survey to upload the QSF file(s) into Qualtrics. After selecting *Create a New Project,* select “Survey” from under the “From Scratch” section.
3. Title the project with the name of that specific IAT, (i.e., “Female Gender Identity IAT” or “Male Gender Identity IAT”). Under the drop-down question “How do you want to start your survey?” *Import a QSF File* should be selected.
4. Labeling the blocks within each IAT survey is recommended to streamline transferring IATs to the created survey later (e.g., Sci_1, Sci_2, Sci_3, Sci_4).
5. Save this IAT survey to the folder created in Step 1.
6. Repeat steps 2–4 for each remaining IAT, creating each IAT as its own survey. Following our example, we would have 11 separate IAT surveys.
7. Once all the IATs are uploaded into Qualtrics as separate surveys, they can be transferred into a pre-existing survey.
8. To do that, go to the existing survey or one of the new IAT surveys, *create a new block* → “Import from Library” → “Copy from the Existing Survey,” and then find the survey created with the IAT from the QSF file and import the IAT blocks. There should be four blocks for each IAT, with a total of 28 questions for each IAT. Each of the four blocks must be imported into their own blocks; otherwise, all 28 questions will be imported into a single block. So, there should be four blocks of seven questions each (see Fig. 4 for this process).

#### Survey flow

The survey flow is the most crucial part of establishing proper survey functionality for multiple IATs. As the survey flow functionality in Qualtrics can get complex quickly, we urge researchers to move slowly through this section and to conduct rigorous testing to ensure the survey flow and presentation of IATs conform to their plans. The survey flow is where researchers assign the IATs to be dynamic and randomly presented to participants. This section will provide instructions on structuring the survey flow. We recommend users review the supplemental materials while implementing changes in their survey flow to reinforce concepts covered (see SOM 1).

##### Multiple and dynamic IATs through survey flow

There are two crucial elements to successfully implementing multiple, dynamic IATs: *1)* setting the *embedded data* in Qualtrics, and *2)* correctly utilizing the randomization features between and within blocks for each IAT.

In our Balanced Identity Design example, we study the implicit STEM identities of participants across three STEM majors, male and female gender identities, and the implicit associations between participants’ gender and STEM major. To achieve this, we develop two gender identity IATs, three STEM major IATs (engineering, science, and computer science), and six IATs, each representing one of the gender-major combinations.

#### Embedded data

**Fig. 5:**
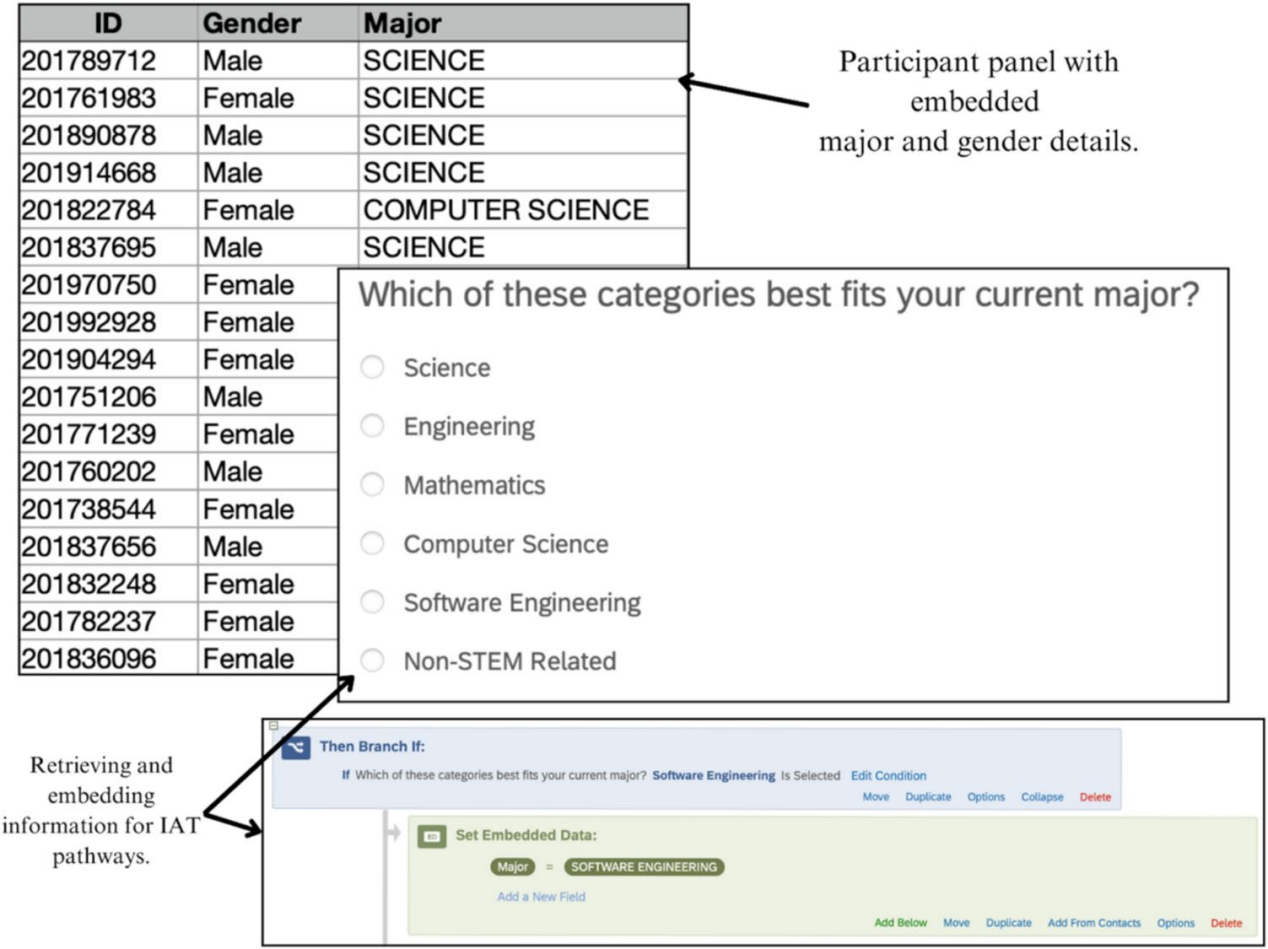
Two ways to embed data for IAT pathways

Embedded data is information recorded in survey data beyond question responses (Qualtrics, 2023). Using embedded data helps dynamically present the correct IATs based on a participant’s profile and responses to questions about their gender identity and STEM major, in this instance. Additionally, embedded data can be preprogrammed parameters based on data known to the researchers prior to the study, such as in pre-post or longitudinal research designs. In our example, by embedding the variable “MAJOR” in the Qualtrics survey, participants’ selection of their major can be recorded as a response to the item and used to conditionally direct them to one of three IATs corresponding to their major. The same applies to participants’ “GENDER” identity. Extending this example, a participant who identifies as a female science major would have those two pieces of data either hard coded into the study distribution list (see the top left of Fig. 5) or pulled from responses to Qualtrics survey items (see bottom right of Fig. 5), which would be used as the pathway to direct the participants to the appropriate three (out of a possible 11) IATs. These two data points would be embedded in the final survey dataset as MAJOR = Science and GENDER = Female. See online supplemental materials for further instructions on programming embedded data in Qualtrics (see SOM 1).

#### Randomization

In addition to embedded data, the randomization features of the survey flow between IATs and the blocks within each IAT are crucial. It is important to present the IATs randomly, and to randomize the IAT blocks within each IAT to reduce the risk of order effects (e.g., Greenwald et al., 2022). The survey flow feature of Qualtrics can evenly and randomly present the blocks within each IAT to ensure equal counterbalancing. Counterbalancing the block order between subjects has been found to eliminate order effects (Messner & Vosgerau, 2009), essential for drawing meaningful interpretations of the data.

To record the sequence in which the IATs were shown to a participant, multiple “branch if” in the survey flow are created. This “branch if” determines if the first question of each of the four blocks of the other IATs has *not* been displayed. For example, if the gender IAT is shown first to a participant, the “if statement” will check if the STEM or STEM-Gender association has *not* been displayed. The embedded data of “first seen,” "second seen," and "third seen" (in the case of three IATs) is recorded for this purpose. In our example, the IATs may be presented in the following order:

1. Female-Science Association IAT (FirstSeen = Association)
2. Female Gender Identity IAT (SecondSeen = Gender)
3. Science Identity IAT (ThirdSeen = STEM)

**Fig. 6:**
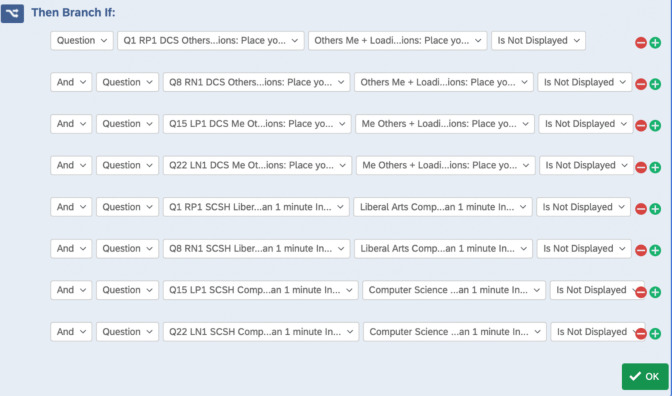
Screenshot of branches for recording the sequence seen. *Note.* Questions 1, 8, 15, and 22 are the first questions in each block. Therefore, the first seen embedded data is set to Gender if those questions are not seen

**Fig. 7:**
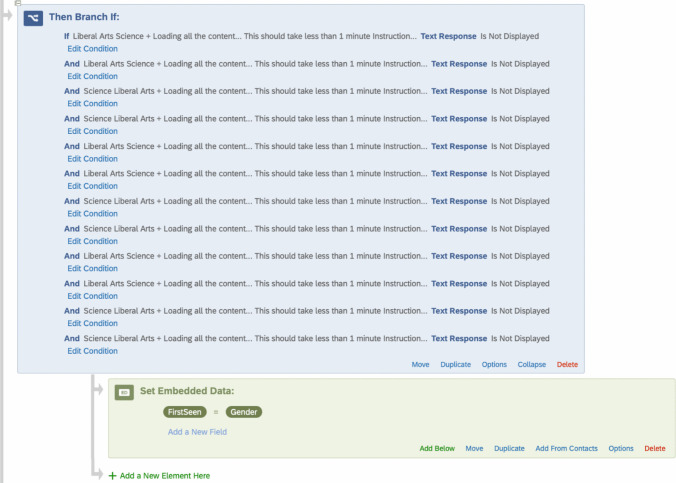
Final example of embedding IAT sequence

This process is repeated to determine the first, second, and third seen for each participant. See Figs. 6 and 7 and SOM 1 for the complete guide.

#### E-mail triggers

Participants do have a natural tendency to attempt to complete their survey on a mobile device. In Qualtrics, you can use the e-mail trigger function to alert the research team if a participant tries to complete the survey on a mobile device. Then, the research team can send the participant a new survey link to complete. The process of implementing the e-mail trigger is as follows:

1. Open the “Survey Flow” tab in the survey selected.
2. Locate the branch that ends the survey with a reminder message not to take the survey on a mobile device.
3. On the “End of Survey” block, select “Customize” and choose “Custom end of survey message…” and then pick a created message that informs them that a new link will be sent to them. (Make sure this message is created beforehand).
4. Then, open “Survey Options” and select “Post Survey.”
5. Under “Manage E-mail Triggers,” select “Edit Triggers,” then select “Add a condition.”
6. Set the condition to “Question” → “No mobile device message” → “is displayed.”
7. Then, complete the e-mail fields.
  - To: Send the trigger to whoever will be responsible for sending new links.
  - Subject: “New Link Needed”
  - Message: ${m://ParticipantID}-${m://Email} has attempted to take the survey on their mobile device. Please send them a new link.” Please note that “ParticipantID” and “Email” will be the unique names you have labeled those variables in your study.
  - When: Immediately
8. Once this has all been completed, *publish* the survey or the survey will not update.
9. Before you send the new link to the participant, ensure you delete their first, attempted response.

#### Testing

At this point, the researcher has set up the Qualtrics survey and is ready to test the selection and flow of the IATs before beginning live data collection with participants. We urge researchers to carefully note any errors encountered and methodically backtrack through the survey flow, the embedded data, and the survey items or list components that trigger the IAT flow. Once the researchers are happy with the flow of the IAT presentation, test the IAT backend data to check that it is recording correctly.

### Step 3: IAT data

#### Exporting data

This section highlights how to download data for analysis and verify that data are being recorded correctly. To initiate data processing after live data collection or testing, export the data from Qualtrics to a.csv file that the researcher can save on their computer, in the cloud, or on a server.

Follow these steps in Qualtrics:

1. Create a folder on your computer to store the R code and the.csv IAT dataset.
  - Add the R code to the folder
2. To download the IAT data, navigate to the survey containing the data you wish to analyze.
3. Access Data & Analysis
4. Select Export & Import
5. Select Export Data
6. Select.csv file type
7. Use choice text
8. Select Download

The.csv file will be downloaded to your device. To enable proper data import in RStudio, ensure the.csv file and the intended R code are in the same folder within your working directory.

The IAT data must be scored to produce a D-score for each participant for each IAT (see Greenwald et al., 2003 for the scoring algorithm). Carpenter et al. (2019) provide an R code file to analyze data from individual IATs downloaded from Qualtrics. The R script and package provided are equipped with features that include data cleaning and analysis following the procedures of Greenwald et al., (2003). However, all the parameters can be customized for each user. The authors provide R scripts to simplify data analysis for users with multiple IATs and include built-in help documentation that provides more information about the analysis functions.

**Fig. 8:**
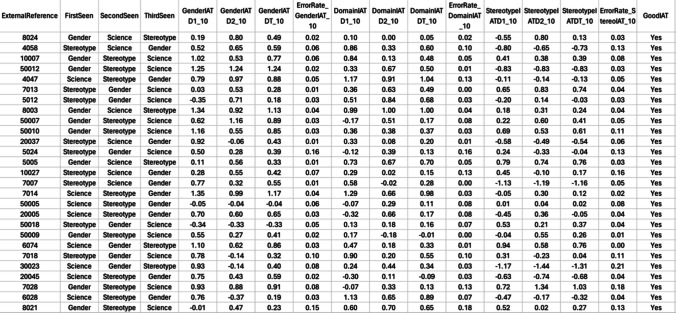
Example of cleaned IAT data processed using Carpenter et al. (2019) R script

1. First, install the iatgen R package (code can be found here: https://osf.io/ew2gn).
2. Once installed, the R code to be used has already been written for one IAT by Carpenter et al. (2019) found here: https://osf.io/sdgba). The R code provided in SOM 3 has adapted for multiple IATs.
3. Within the sample code provided by the authors, users should change the variable names within each IAT to match those labeled in the downloaded Qualtrics files. Upon completion, a new data file containing the D-scores for each participant, as well as the “First Seen,” “Second Seen,” and “Third Seen” are output into a spreadsheet for analysis (see Fig. 8).

Carpenter and colleague’s (2019) resources are innovative, and we extended their work here to accommodate output for multiple IATs. These specifications include recording the specific IAT randomly presented, the sequence in which the IATs were seen, and tracking the number of correctly completed IATs by the participants, based on recommendations by Greenwald et al. (2022). These additional specifications play a crucial role in enhancing the validity and reliability of findings, consequently contributing to the credibility of the research outcomes (see SOM 3 for supplemental R code).

#### Testing procedures

One of the most important procedures in creating multiple, dynamic, and simultaneous IATs is extensively testing each condition before live data collection. We encourage, if possible, gathering a group of associates and assigning different conditions to each to ensure each IAT is functioning appropriately. There are a few key things to look out for while testing. First, ensure the proper conditions are directed to the corresponding IATs and relevant questions. Also, check if participants respond to the IAT on a computer/laptop, not a tablet or mobile device. Second, export the data and ensure the embedded data was saved correctly throughout the survey. In this step, we also run the R-code to ensure that D-scores are accurately calculated.

## Section 2: Empirical example

The empirical example here draws upon the Balanced Identity Design (Greenwald et al., 2002) as a theoretical framework for researching racial/ethnic diversity in STEM.

### Overview

This study uses Qualtrics survey-based IAT data to test hypotheses about racial/ethnic identity balance in undergraduate STEM majors as they approach graduation. Balanced Identity Design Theory posits that two central identities can be classified as *balanced* or *imbalanced* within the context of stereotypic associations between the two identities (Bruni et al., 2021; Cvencek et al., 2012; Greenwald et al., 2002; Woodcock et al., 2025). Prevailing social stereotypes describe and prescribe which identities are compatible and for whom; for example, racial/ethnic stereotypes typically favor Whites and Asians in STEM. Therefore, understanding how individuals integrate a new, central identity, such as a STEM identity, in the face of negative stereotypes may be critical to issues of identity and underrepresentation. Using BID to understand imbalanced and balanced identities is critical to issues of identity and underrepresentation, as whether one’s identities psychologically align can relate to a decline in intention to pursue STEM. For example, one study found that novice STEM majors from negatively stereotyped groups were less likely to hold identity balance than their positively stereotyped counterparts, while students (in a different sample) approaching graduation achieved identity balance, demonstrating that identity balance can serve as a psychological mechanism for supporting persistence and making BID valuable for addressing issues of identity and underrepresentation (Woodcock et al., 2025).

This study sought to identify potential relationships between racial/ethnic and STEM domain identities and race/ethnicity-STEM associations measured explicitly via validated survey scales and implicitly via IATs. More specifically, the study aimed to understand *1)* how the implicit measures of identity might correspond with their counterparts measured at the explicit or conscious level, and *2)* if this relationship was different for White and Hispanic students in STEM. Given that the implicit and explicit measures of identities and associations are related but distinct constructs (Greenwald et al., 2002; Hofmann et al., 2005a, 2005b), we hypothesized that there will be a small positive correlation between each of the implicit racial/ethnic identity (me = my race/ethnicity^5^) and STEM identity (Me = my STEM major) and their explicit counterparts for both White and Hispanic students. We hypothesize that the pattern of correlations between explicit and implicit racial/ethnic-STEM associations differs for White and Hispanic students. Hispanics in the U.S. face negative stereotypes about their ability to perform well in many STEM fields (Massey & Fischer, 2005; Woodcock et al., 2012), and explicit measures are susceptible to impression management, so we expect a divergent correlation between Hispanic students’ explicit endorsement of the STEM = White association and their implicit associations compared with their White peers. It is likely that Hispanics in STEM fields would explicitly endorse the notion that STEM = Hispanic but harbor STEM = White implicit associations that are aligned more with social stereotypes.

## Method

### Participants

These analyses are drawn from data from a longitudinal study of 1310 White and Hispanic STEM majors from 12 West Coast state universities. Data were collected over five years beginning in the Fall of 2019. The analytic sample included 1003 participants with complete IAT data (three IATs with error rates of < 30%) and who had responded to the Fall 2020 wave of the longitudinal survey. The analytic sample was 55% Hispanic/Latino and 45% White, 51% women, with a mean age of 22.8 years. Participants were declared STEM majors in Biological and Life Sciences (47%), Engineering (33%), Mathematics (5%), and Computer Science (15%). Twelve percent were Juniors, 72% were seniors, and 16% were 5th year seniors or “other.”

### Procedure

An initial screening Qualtrics questionnaire was e-mailed to lists of White and Hispanic/Latino(a)^6^ declared STEM majors in their Junior or Senior year across 12 university campuses. Eligible participants were invited to a 5-year longitudinal study called *My College Pathways*. Participants were e-mailed a link to complete IAT and survey data twice per academic year from 2019–2024. Participants completed three randomly displayed, personalized online IATs (Greenwald et al., 1998), and then a battery of survey items. Data for these analyses includes explicit measures of STEM identity, racial/ethnic identity, and the endorsement of STEM-racial/ethnic stereotypes.

### Measures

#### Explicit measures

##### Explicit STEM identity

Explicit STEM identity was measured using an 11-item scale adapted from the Science Career Identity Scale (Chemers et al., 2011). The scale measured participants’ perceptions of how their identity aligns with their selected major (i.e., Computer Science, Engineering, Science). Items were measured on a scale of 1 (strongly disagree) to 5 (strongly agree) . Items included: “*Being a scientist is an important reflection of who I am*”. The scale items were averaged to produce one STEM identity score, which exhibited acceptable reliability (*α* =.88).

##### Explicit race/ethnic identity

To measure the degree to which the participant identifies with their stated race/ethnicity, a six-item ethnic identity scale, the Multigroup Ethnic Identity Measure – Revised (MEIM-R: Phinney & Ong, 2007) was presented (e.g., “*I have a strong sense of belonging to my own ethnic* group” or “*I understand pretty well what my ethnic group membership means to* me”). Scale items were rated on a scale of 1 (strongly disagree) to 5 (strongly agree). A scale score was developed by averaging the item responses, which exhibited acceptable reliability (*α* =.92).

##### Explicit STEM stereotype endorsement

To measure the extent to which each participant endorses racial/ethnic stereotypes associated with their STEM major, the STEM Stereotype Endorsement scale (Schmader et al., 2004) was used (e.g., *“In general, White students may be better at [STEM major]”*)*.* This two-item scale was measured from 1 (strongly disagree) to 7 (strongly agree), and each scale item was tailored towards the participant’s STEM domain (engineering, science, or computer science). A composite score was calculated in which higher scores indicated a greater endorsement for STEM = White racial/ethnic-STEM stereotypes, which exhibited acceptable reliability (*α* =.76).

#### Implicit measures

Three IATs measured implicit racial/ethnic identity, STEM identity, and STEM-race/ethnic associations. The IATs were STEM major-specific for each participant. For example, engineering students responded to IATs that reflected their engineering identity, their ethnic identity, and their engineering-ethnicity associations (see Table 1 for an example category and stimuli and see SOM 4 for all categories and stimuli). IAT D-scores were calculated using the algorithm outlined by Greenwald and colleagues (2003). IAT D-scores range from approximately – 2 to 2, with scores from zero to |0.2| denoting small, scores from |0.2 to 0.5| denoting moderate, and scores greater than |0.8| denoting large implicit associations. Each of the IAT measures had normally distributed data and we verified that our data met the balanced identity design criteria using a series of regression analyses outlined by Greenwald et al. (2002). (See SOM 5 for histograms and SOM 6 for the full set of aggregate balanced identity regression analyses).

The analyses included in this manuscript are for illustrative purposes – the study was not preregistered.

## Results

The means and standard deviations by race/ethnicity are featured in Table 2 (See SOM 7 for means and standard deviations of implicit and explicit measures by race/ethnicity and major).

**Table 1:** Categories and stimuli for IATs

	Race/ethnicity identity							
Categories	*Me*	*Other*		*White*		*Hispanic*		
Stimuli	Me	Their		Anderson		Mendez		
	Myself	Them		Thomas		Perez		
	I	They		Smith		Rojas		
				Miller		Escobar		
				Moore		Fernandez		
	STEM identity							
Categories	*Me*	*Other*		*Computer Science*			*Liberal Arts*	
Stimuli	Me	Their		Programmer			Literature	
	Myself	Them		Algorithm			Arts	
	I	They		Device			Philosophy	
				Software			Music	
				Code			History	
	Race/ethnicity-STEM associations							
Categories	*White*		*Hispanic*		*Computer Science*			*Liberal Arts*
Stimuli	Anderson		Mendez		Programmer			Literature
	Thomas		Perez		Algorithm			Arts
	Smith		Rojas		Device			Philosophy
	Miller		Escobar		Software			Music
	Moore		Fernandez		Code			History

Computer Science is used as the example of STEM identity and gender-STEM IATs

Implicit and explicit STEM identity (specific to individual major) were correlated (with 1000 bootstrapped samples^7^) separately for the White and Hispanic participants (*r* =.21**^8^ and *r* =.09*, respectively). The correlations were significantly different from zero and marginally significantly different from one another (*z* = 1.92, *p* =.055). This suggests that implicit and explicit STEM identity are more independent constructs for Hispanic students.

Implicit and explicit ethnic/racial identity were correlated separately for the White and Hispanic participants (*r* =.10 and *r* =.12**, respectively) and were found to be small but significantly different from zero for both White and Hispanic students, and not significantly different from one another (*z* = 0.31, *p* =.75). This suggests that implicit and explicit racial/ethnic identity are separate but related constructs.

Last, we correlated implicit and explicit STEM-race/ethnicity associations (specific to individual majors and race/ethnicity) separately for the White and Hispanic participants. Among White participants, implicit My Ethnicity = STEM D-scores were not significantly correlated with explicit My Ethnicity = STEM scores (*r* =.007), and the correlation among Hispanic participants were also not significantly different from one another (*z* = 1.15, *p* =.25). This suggests that these are separate constructs for both White and Hispanic students.

**Table 2:** Means and standard deviations for implicit and explicit measures by race/ethnicity

	White		Hispanic	
Variable	*M*	*SD*	*M*	*SD*
Implicit measures				
STEM Identity (IAT)	.27	.38	.39	.35
Race/Ethnic Identity (IAT)	.26	.37	.32	.38
Race/Ethnicity-STEM Associations (IAT)	–.02	.49	.36	.46
Explicit measures				
STEM Identity^	3.71	0.79	3.71	0.80
Explicit Race/Ethnic Identity ^	2.53	1.06	3.57	0.99
Explicit STEM Stereotype Endorsement ~	2.89	0.98	3.13	1.08

^ denotes 1–5 scale. ~ denotes 1–7 scale

## General discussion

This methodological expansion extends the Carpenter et al. (2019) paper by presenting a method for developing and implementing multiple, dynamic IATs within a single Qualtrics survey. The step-by-step instructions first walk researchers through building multiple IATs using Carpenter et al.’s (2019) method, then provide our own guidance for programming the IATs into Qualtrics, presenting them dynamically and randomly, and setting up backend information for data analysis. Additionally, we provide R code to calculate IAT accuracy and record the IAT order of encounters, both of which are vital to improving data validity. IAT accuracy identifies whether IATs are being completed properly, while recording and randomizing encounter order guards against order effects, which have been found to influence IAT data (Messner & Vosgerau, 2009).

The empirical example in this paper shows that the multiple IAT method can be effectively implemented. Participant-tailored and dynamically randomized IATs for racial/ethnic and STEM identities, as well as their associations, were successfully integrated into a single Qualtrics survey without affecting data validity. This method supports larger-scale, theory-driven research and is a valuable tool for researchers needing multiple simultaneous IATs. Overall, our extension is a valuable contribution as it provides researchers with both the guidance and materials needed to properly build IATs, program Qualtrics, and analyze IAT data, as even small mistakes when using multiple IATs can introduce serious data validity issues.

### Future directions

Methodologically, the expansion of improving online IATs, by including multiple dynamic IATs has enhanced the method but is still a work in progress. Currently, Qualtrics IATs powered by iatgen are incompatible with mobile and tablet devices, limiting iatgen IATs use to desktop and laptop computers. To address this limitation, a crucial improvement would be to make the iatgen online survey software IATs mobile and tablet-friendly. Given the significant increase in reliance on mobile and tablet devices, developing a multimodal format will be imperative for the effective use of this method. Fortunately, scholars have already begun to address, this issue. For instance, Cui and colleagues (2021) have developed an open-source web app for creating mobile-friendly Qualtrics-based IATs. Additionally, while the current work addresses a significant need for implementing dynamic, multiple IAT surveys, future work can continue to modify procedures and R Scripts to be more efficient and easily adaptable for users with various coding experience. Finally, given that Qualtrics is a commercial platform requiring payment and is not accessible to all researchers, continued development of flexible and accessible tools remains essential, with developments in the works. Those without Qualtrics can use available free tools for running online studies to implement IATs such as Data Pipe or Minno.js.

## Data Availability

The data and materials are available on OSF repository—https://osf.io/b4jkc/, and the study was not preregistered.

## Footnotes

1 https://iatgen.wordpress.com is the original website with additional resources. The http://iatgen.org/ webpage is within the original website that is used to create IATs.

2 Science, engineering, and computer science are STEM majors.

3 IATs 1 and 2 = Gender Identity, IATs 3 to 5 = STEM Identity, and IATs 6 to 11 = Gender – STEM Associations.

4 While the relative validity and reliability of different stimuli are beyond the scope of this paper, it can be noted that there is a growing body of empirical literature that can guide the relative strengths and weaknesses of different IAT stimuli for specific purposes (e.g., Axt et al., 2021). Greenwald and colleagues’ excellent paper on IAT best practices also devotes two large sections to exemplar and stimuli selection (Greenwald et al., 2022).

5 As identified by participants.

6 The term “Hispanic/Latino(a)” is used here to reflect the language of the original study. However, terminology in this area has evolved, and more inclusive terms like “Latinx” or “Latine” are more often preferred.

7 To account for non-normality of the explicit identity data.

8 * Denotes significance at *p* =.05, and ** denotes significance at *p* =.01.

## Code availability

Available on OSF repository—https://osf.io/b4jkc/
